# Association between fentanyl use and reduced risk of tension pneumothorax in extremely preterm infants born at 22–23 weeks' gestation: a retrospective case–control study

**DOI:** 10.3389/fped.2025.1643333

**Published:** 2025-10-03

**Authors:** Tomonori Kurimoto, Tokuhisa Takuya, Hiroshi Ohashi, Eiji Hirakawa, Masaya Kibe, Takatsugu Maeda, Masato Kamitomo

**Affiliations:** Department of Neonatology, Perinatal Medical Center, Kagoshima City Hospital, Kagoshima, Japan

**Keywords:** tension pneumothorax, extremely preterm infants, fentanyl, 22–23 weeks of gestation, the first 72 h of life

## Abstract

**Background:**

Tension pneumothorax is a life-threatening complication in extremely preterm infants, especially within the first 72 hours of life. Identifying preventive strategies is crucial to improving survival rates. This study aimed to evaluate the association between fentanyl use and the incidence of tension pneumothorax in extremely preterm infants born at 22–23 weeks of gestation.

**Methods:**

This retrospective case–control study was conducted at a tertiary care center in Japan. It included 138 preterm infants with a gestational age from 22 weeks + 0 days to 23 weeks + 6 days (January 2010 to March 2023). Logistic regression and propensity score matching were used to adjust for confounding factors such as chorioamnionitis stage 2–3, oligohydramnios, antenatal steroids, ventilation mode, persistent pulmonary hypertension, and birth at a primary or secondary perinatal center.

**Results:**

Sixteen infants (11.6%) developed tension pneumothorax within 72 h of birth, with a mortality rate of 68.7%. The use of fentanyl was associated with a reduced risk of tension pneumothorax (adjusted odds ratio: 0.1; 95% confidence interval: 0.01–0.75).

**Conclusion:**

The use of fentanyl may reduce the risk of tension pneumothorax within the first 72 h of life in infants born at 22–23 weeks of gestation. Further randomized controlled trials are needed to validate these findings and assess long-term outcomes.

##  Introduction

Tension pneumothorax is a life-threatening complication in extremely preterm infants. The incidence of pneumothorax in preterm infants born at 23–28 weeks of gestation within 72 h is reportedly 9.2% (1). In our institution, the incidence of tension pneumothorax after birth in infants born at 22–23 weeks of gestation was higher at 14.1%, with 71.4% occurring within 72 h after birth. This condition was identified as a significant risk factor for mortality, with an odds ratio (OR) of 5.88 (95% confidence interval [CI]: 1.57–22.0) (2). It remains unclear whether antenatal corticosteroid administration affects the risk of pneumothorax (relative risk [RR]: 0.76, 95% CI: 0.32–1.80) (3). Although surfactant replacement therapy has been shown to reduce the incidence of pneumothorax, extremely preterm infants born at 22–23 weeks of gestation remain at a disproportionately high risk of developing tension pneumothorax (4). This highlights the need for additional strategies to further reduce the incidence of this life-threatening complication.

Despite advances in neonatal care, including the use of antenatal corticosteroids and surfactant therapy, the mechanisms underlying the increased incidence of tension pneumothorax in this population remain poorly understood. Furthermore, there is a lack of comprehensive studies investigating modifiable risk factors and evaluating potential therapeutic approaches specifically targeting this vulnerable group.

In clinical practice, sedation and analgesia are often administered to facilitate mechanical ventilation in extremely preterm infants, including those born at 22–23 weeks of gestation. However, evidence supporting their role in reducing the risk of pneumothorax is limited, and their potential impact remains unclear.

Given these gaps in knowledge, this case-control study aimed to identify risk factors for pneumothorax in preterm infants born at 22–23 weeks of gestation within the first 72 h after birth. By addressing these uncertainties, we hope to guide clinical practices and develop targeted strategies to improve outcomes in this high-risk population.

## Methods

### Patients

Informed consent was obtained from the parents or guardians of all enrolled participants. Furthermore, this retrospective case-control study adhered to the principles outlined in the Declaration of Helsinki, as revised in Tokyo in 2004. This study was approved by the Ethics Committee of Kagoshima City Hospital (Approval No. 2023-22). This study was conducted at Kagoshima City Hospital, the only tertiary care center in Kagoshima Prefecture, Japan. Our hospital serves as a regional referral center, managing maternal transfers from primary and secondary perinatal institutions and accepting patients expected to deliver at 22–23 weeks of gestation. In cases where prenatal maternal transfer was not feasible, newborns delivered at primary or secondary perinatal institutions were transferred postnatally. For these cases, a neonatologist was dispatched to the delivering facility, provided resuscitation, and subsequently transported the newborn to the neonatal intensive care unit (NICU) using specialized neonatal transport vehicles. Data from consecutive cases were collected using File Maker Pro (database management system, Claris International Inc., Santa Clara, CA, USA). This study included live-born infants with a gestational age (GA) of 22 weeks + 0 days to 23 weeks + 6 days, born between January 2010 and March 2023. We retrospectively compared 16 cases of tension pneumothorax with 122 cases of non-tension pneumothorax.

### Data collection

The GA was determined using early pregnancy ultrasound data, either based on the last menstrual period or crown-rump length, or a combination of both. Between January 2010 and March 2023, a total of 140 infants born at 22–23 weeks of gestation underwent resuscitation. Two patients with congenital malformations, including tetralogy of Fallot with pulmonary atresia and severe pulmonary stenosis, were excluded from the study. Abnormal fetal heart rate prior to delivery was evaluated in accordance with the 2009 recommendations of the National Institute of Child Health and Human Development (5–7).

### Initial treatment

The postnatal blood pressure of the infants were monitored using either a 2.5 or 3.5 Fr umbilical artery catheter or a 24G peripheral arterial line. The umbilical artery catheter was removed between postnatal days 3 and 5 and replaced with a peripheral arterial line, maintained for 28–35 days post-birth. Blood sampling was conducted every 6–8 h to adjust the infusion contents and manage respiratory status during the first week. The frequency of endotracheal suctioning during the first 2 weeks postnatally was 6–9 times daily.

Central body temperature was monitored using a temperature probe (Dräger, Lübeck, Germany) placed on the infant's back. Peripheral body temperature was assessed with a probe placed on the sole of the infant's foot. Care was provided with minimal handling to reduce stress, and continuous monitoring of body temperature, blood pressure, oxygen saturation, end-tidal CO_2_, and heart rate was maintained. Humidity and temperature in the incubator were regulated according to standardized protocols (8).

### Ventilation

All infants were intubated in the resuscitation room because of respiratory distress syndrome or apnea risk, requiring FiO_2_ ≥ 0.4 to achieve the target peripheral SpO_2_ (oxygen saturation measured by pulse oximetry), followed by surfactant administration. We typically began using a synchronized mode with mandatory and spontaneous breathing (SIMV + PS). Volume-targeted (VT) ventilation with 4–6 ml/kg, positive end-expiratory pressure (PEEP) between 4.5 and 6.0 cmH_2_O, and inspiratory time (Ti) in the range of 0.3–0.4 s were used. Continuous SpO_2_ monitoring and end-tidal CO_2_ (EtCO2) measurements were performed. The volume guarantee (VG) mode was not used immediately after birth; however, if there was no bilateral lung field opacity on radiography, no atelectasis, or no pneumothorax or if the proper placement of the endotracheal tube at the level of the second intercostal space was confirmed, a switch to the VG mode was made. The VG mode was set to 4–6 ml/kg. Despite the SIMV mode settings at a peak inspiratory pressure of 20–21 cmH_2_O, PEEP of 6 cmH_2_O, and respiratory rate of 60–65/min, respiratory acidosis with arterial blood gas findings of pH <7.2 and PaCO_2_ >65 mmHg were maintained (9). In cases where the response to SIMV was deemed insufficient, as indicated by the inability to decrease PCO_2_ by >10% and/or FiO_2_ by >20% within 1 h of initiating SIMV, we opted for transition to high-frequency oscillatory ventilation (HFOV) (i.e., rescue HFOV) (10).

The mean airway pressure (MAP) was 2–3 cmH_2_O above that of the SIMV but had to be sufficient to inflate the lungs (i.e., with complete white-out and stiff lungs, a markedly higher MAP was required).

A frequency range of 12–15 Hz and an inspiration:expiration ratio of 1:1 were used. If adequate PaCO_2_ was not achieved at the maximum amplitude, the frequency was decreased to 10 Hz (11). The use of VT was managed within the target range of 1.5–2.0 ml/kg (12).

### Diagnosis and treatment of tension pneumothorax

Radiographic examination revealed displacement of the affected lung away from the chest wall. Other diagnostic indicators included diaphragmatic depression, contralateral mediastinal shift, and transillumination along the posterior axillary line in suspected lateral luminescent tension pneumothorax cases. The diagnosis of tension pneumothorax was made by at least two neonatologists based on both clinical symptoms (respiratory distress and circulatory instability) and chest radiographic findings. Treatment involved fine-needle aspiration followed by chest tube insertion. We focused specifically on tension pneumothorax rather than pneumothorax in general, because tension pneumothorax represents a severe condition requiring urgent chest tube drainage. It is associated with significant hemodynamic compromise, which increases the risk of intraventricular hemorrhage (IVH) and mortality in neonates, thereby having greater clinical relevance compared with non-tension pneumothorax.

### Persistent pulmonary hypertension of the newborn

Echocardiographic evidence of persistent pulmonary hypertension of the newborn (PPHN) was defined as an estimated peak systolic pulmonary artery pressure of 35 mmHg or more, which is greater than two-thirds of the systemic systolic pressure. The evaluated indicators included a tricuspid regurgitation jet, right-to-left patent ductus arteriosus shunt, or right-to-left shunt observed at the arterial level (13).

### Oligohydramnios

Two ultrasonographic modalities are commonly used to estimate the amniotic fluid volume: the amniotic fluid index (AFI) and the maximum vertical pocket technique (MVP).

Oligohydramnios was diagnosed when the AFI was <5 cm or the MVP was <2 cm (14, 15).

### Chorioamnionitis

Pathological placental findings included chorioamnionitis (CAM) and funisitis. Based on Blanc's classification, CAM was staged according to the extent of polymorphonuclear leukocyte infiltration, with stages 1, 2, and 3 indicating infiltration into the intervillous space, chorion, and amnion (16), respectively.

### Fentanyl

Fentanyl was administered in cases of PPHN and intraventricular hemorrhage (IVH). Since September 2019, fentanyl has been administered to prevent IVH after confirming the absence of significant hypotension upon admission. The administration of fentanyl (1.0–2.0 μg/kg/h) was started after catheter insertion to 72 h after birth (17–20). Fentanyl was administered prior to the occurrence of tension pneumothorax. Before 2019, fentanyl was also used in some cases; however, phenobarbital was the predominant sedative, and midazolam or dexmedetomidine were frequently administered as concomitant medications.

### Statistical analysis

Categorical variables were compared between the two groups using the chi-square test or Fisher's exact test, as appropriate. Continuous variables were compared using the Mann–Whitney *U* test. To evaluate the effect of fentanyl administration on the incidence of tension pneumothorax, propensity score matching was performed to adjust for potential confounding factors. Propensity scores were estimated using a logistic regression model, with fentanyl administration as the dependent variable. Independent variables included prenatal factors such as CAM stage 2–3, oligohydramnios, and antenatal steroid administration; postnatal factors such as PPHN and ventilation modes (SIMV, HFOV, and VG mode); and institutional factors, including birth at primary or secondary perinatal centers.

Matching was performed using the nearest-neighbor method with a 1:1 ratio, and caliper widths of 0.2 and 0.3 standard deviations (SD) were tested to ensure consistency. The balance between matched groups was evaluated using standardized mean differences (SMDs), with an SMD < 0.1 indicating an adequate balance.

To further examine the robustness of the findings, sensitivity analyses were conducted. These included multivariate logistic regression models to adjust for residual confounding factors and Firth logistic regression analysis to address potential bias caused by rare events.

Subgroup analyses were performed to evaluate variations in the effect of fentanyl use in specific high-risk populations. Analyses were conducted for infants with PPHN, infants born at primary or secondary perinatal centers, and infants with CAM stage 2–3. For variables with limited sample sizes (e.g., VG mode), multivariate logistic regression models were used to account for residual confounding.

Missing data were handled using a listwise deletion approach, and statistical significance was defined as *p* < 0.05.

### Additional analyses

To further investigate the relationship between fentanyl administration and tension pneumothorax, additional analyses were conducted.

A total of 54 neonates received fentanyl for sedation or pain management in the NICU. Among these neonates, only one (1.85%) developed tension pneumothorax within 72 h after birth. Given the rarity of this outcome, Firth logistic regression analysis was employed to mitigate potential bias caused by complete separation. Analyses were performed separately for primary and secondary variables, and covariates were included in all models to adjust for confounding effects.

The primary outcome of this study was the incidence of tension pneumothorax within 72 h after birth. Key variables related to fentanyl administration were assessed, including infusion rate (μg/kg/h, continuous), duration of administration (hours), and high total fentanyl dose (≥140 μg/kg/day). Secondary factors such as ventilation mode changes and surfactant re-administration were also analyzed. Additional covariates included PPHN, ventilation modes (SIMV, HFOV, VG mode), birth location, oligohydramnios, antenatal steroid administration, and CAM stage 2–3.

In addition to the primary case–control analysis, we performed a cohort-style sensitivity analysis stratified by fentanyl exposure within 72 h after birth. To avoid immortal time bias, infants were considered exposed only if fentanyl was initiated before any TP event. Confounding was addressed with inverse probability of treatment weighting (IPTW) based on a propensity score including key prenatal, postnatal, and institutional factors. Balance was assessed by SMD (SMD < 0.1), and visually inspected using a Love plot. The primary outcome was TP within 72 h; secondary outcomes were mortality before discharge and severe IVH. Sensitivity analyses were conducted using multivariate or penalized logistic regression (Firth method), and propensity score–based approaches [IPTW, overlap weighting, augmented inverse probability weighting (AIPW)]. All statistical analyses were performed using R (The R Foundation for Statistical Computing, Vienna, Austria) and JMP 14 (SAS Institute Inc., Cary, NC, USA).

## Results

Of the 138 patients included, 92 (66.7%) were discharged, while 46 (33.3%) died in the hospital. Tension pneumothorax developed in 16 infants (11.6%) within 72 h of birth, with a mortality rate of 68.8% (*n* = 11). Among these, six (37.5%) died within 72 h of birth (Table 1). In the control group, three infants developed tension pneumothorax beyond the early neonatal period with a median onset of 19 days (interquartile range, 16–22.5 days).

**Table 1:** Perinatal and neonatal characteristics of infants with and without tension pneumothorax.

Variable	TP (*n* = 16) % or IQR	Non-TP (*n* = 122)% or IQR	*p* value
22w	6 (37.5)	36 (29.5)	0.57
Sex (male)	7 (43.8)	63 (51.6)	0.6
Birth weight (median)	571 (499–623)	532 (472–652)	0.26
FGR (10%tile)	1 (6.3)	17 (13.9)	0.39
Survival discharge	5 (31.3)	87 (71.3)	0.003
Death (less than 1 week)	6 (37.5)	7 (5.7)	0.001
Non-tertiary hospital birth	3 (18.8)	10 (8.2)	0.18
Maternal age	30 (27–35)	31 (28–34)	0.74
C/S	11 (68.8)	95 (77.9)	0.53
Antenatal steroids	4 (25)	61 (50)	0.07
Tocolysis	10 (62.5)	81 (66.4)	0.78
MgSO4	7 (43.8)	61 (50)	0.79
Antibiotics	3 (18.8)	35 (28.7)	0.56
PROM	7 (43.8)	40 (32.8)	0.41
CAM stage 2–3	9 (56.3)	49 (40.2)	0.28
Funisitis stage 2–3	4 (25)	26 (21.3)	0.75
HDP	0	4 (3.3)	1
GDM	0	5 (4.1)	1
Oligohydramnios	2 (12.5)	4 (3.3)	0.13
Fertility treatment	1 (6.3)	13 (10.7)	1
Abruption	0	13 (10.7)	0.36
Placenta previa	0	1 (0.8)	1
MDtwin	0	10 (8.2)	0.61
APS1min	2 (1–2)	2 (1–3)	0.08
APS 5min	5 (3–6)	6 (5–7)	0.14
UApH	7.27 (7.19–7.35)	7.33 (7.27–7.37)	0.15
RDS	16 (100)	122 (100)	1
IVH Grade 3–4	6 (37.5)	37 (30.3)	0.57
Cystic PVL	0	9 (7.4)	0.6
NEC	0	11 (9)	0.36
FIP	2 (12.5)	17 (13.9)	1
MRI	0	5 (4.1)	1
PDA surgery	1 (6.3)	9 (7.4)	1
EOS	1 (6.3)	2 (1.6)	0.34
PPHN	1 (6.3)	12 (9.8)	1
SIMV	14 (87.5)	110 (90.2)	0.67
HFOV	6 (37.5)	58 (47.5)	0.6
VG	5 (31.3)	56 (45.9)	0.3
Fentanyl use	1 (6.3)	53 (43.4)	0.005
PB	3 (18.8)	35 (28.7)	0.56
DEX	2 (12.5)	9 (7.4)	0.62
MDZ	0	9 (7.4)	0.6
Re-Intubation	1 (6.3)	4 (3.3)	0.47
Cardiac massage	0	2 (1.6)	1
Retreatment of surfactant	2 (12.5)	19 (15.6)	1
Fetal brady cardia	1 (6.3)	8 (6.6)	1

APS, apgar score; CAM, chorioamnionitis; C/S, cesarean section; CI, confidence interval; DEX, dexmedetomidine; EOS, early-onset sepsis; Fertility treatment, including AIH (artificial insemination with husband), ICSI (intracytoplasmic sperm injection), and IVF-ET (*in vitro* fertilization and embryo transfer); FGR, fetal growth restriction; FIP, focal intestinal perforation; GA, gestational age; GDM, gestational diabetes mellitus; HDP, hypertensive disorders of pregnancy; HFOV, high-frequency oscillatory ventilation; IQR, interquartile range; IVH, intraventricular hemorrhage; MDtwin, monochorionic diamniotic twin; MDZ, midazolam; MRI, meconium related ileus; MgSO₄, magnesium sulfate; NEC, necrotizing enterocolitis; PB, phenobarbital; PDA, patent ductus arteriosus; PPHN, persistent pulmonary hypertension of the newborn; PROM, premature rupture of membranes; PVL, periventricular leukomalacia; RDS, respiratory distress syndrome; SIMV, synchronized intermittent mandatory ventilation; TP, tension pneumothorax; UApH, umbilical artery pH; VG, volume guarantee ventilation.

### Baseline characteristics and propensity score matching

The baseline characteristics of the tension pneumothorax and non-tension pneumothorax groups are summarized in Table 1. Following propensity score matching, the baseline characteristics were balanced between fentanyl-exposed and non-exposed groups. SMDs were below 0.1 for all variables except ventilation mode (VG mode: 0.1) and oligohydramnios (0.1) (Table 2, Supplementary Table S1).

**Table 2:** Standardized mean differences after propensity score matching.

Variable	SMD
PPHN	0.08
SIMV	0.08
HFOV	0
VG	0.10
Non-tertiary hospital birth	0.07
Oligohydramnios	0.10
Antenatal steroids	0.04
CAM stage2–3	0.08

SMD, standardized mean difference; PPHN, persistent pulmonary hypertension of the newborn; SIMV, synchronized intermittent mandatory ventilation; HFOV, high-frequency oscillatory ventilation; VG, volume guarantee ventilation; CAM, chorioamnionitis.

### Primary analysis

Logistic regression analysis demonstrated that fentanyl use was significantly associated with a reduced risk of tension pneumothorax (OR: 0.06, 95% CI: 0.01–0.56, *p* = 0.01). Other variables, including PPHN and ventilation modes, showed no significant associations with the outcome.

**Figure 1:**
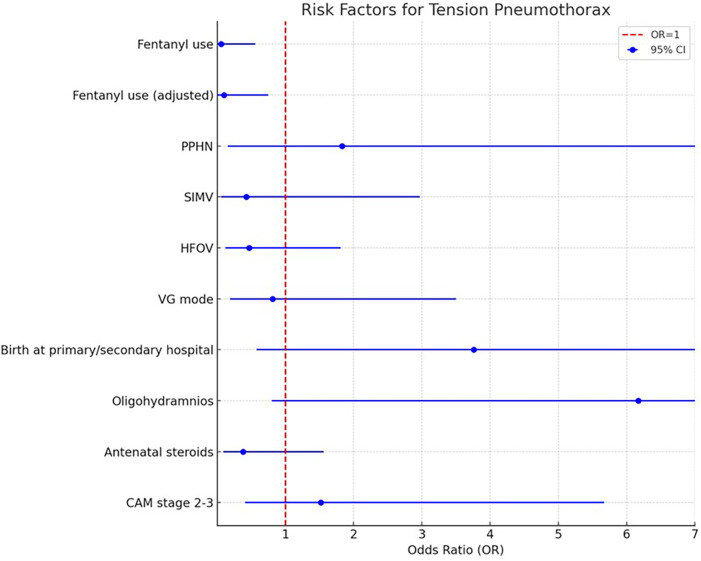
Risk factors for tension pneumothorax.

After adjustment using propensity score matching, fentanyl use remained a significant protective factor against tension pneumothorax (Adjusted OR: 0.10, 95% CI: 0.01–0.75, *p* = 0.03) (Table 3 and Figure 1).

**Table 3:** Subgroup analyses using adjusted logistic regression models.

Variable	Odds ratio (95% CI)	*p*-value
Fentanyl use	0.06 (0.01–0.56)	0.01
Fentanyl use (adjusted)	0.10 (0.01–0.75)	0.03
PPHN	1.83 (0.16–20.7)	0.63
SIMV	0.43 (0.06–2.97)	0.39
HFOV	0.47 (0.12–1.81)	0.27
VG mode	0.81 (0.19–3.50)	0.78
Non-tertiary hospital birth	3.76 (0.58–24.5)	0.17
Oligohydramnios	6.17 (0.8–47.6)	0.08
Antenatal steroids	0.38 (0.09–1.56)	0.18
CAM stage2–3	1.52 (0.41–5.67)	0.54

CAM, chorioamnionitis; CI, confidence interval; HFOV, high-frequency oscillatory ventilation; PPHN, persistent pulmonary hypertension of the newborn; SIMV, synchronized intermittent mandatory ventilation; VG, volume guarantee.

### Subgroup and sensitivity analyses

Subgroup analyses were conducted to further explore the robustness of the results. When infants with PPHN were excluded, fentanyl use remained significantly associated with a lower incidence of tension pneumothorax (Adjusted OR: 0.07, 95% CI: 0.01–0.67, *p* = 0.02). Similarly, excluding cases with CAM stage 2–3, fentanyl use continued to demonstrate a protective effect (Adjusted OR: 0.04, 95% CI: 0.002–0.71, *p* = 0.03).

Analyses limited to births at tertiary care centers (excluding primary and secondary institutions) showed a borderline significant association between fentanyl use and lower tension pneumothorax risk (Adjusted OR: 0.11, 95% CI: 0.01–0.99, *p* = 0.05). Additionally, excluding cases with oligohydramnios preserved the protective effect of fentanyl (Adjusted OR: 0.07, 95% CI: 0.008–0.67, *p* = 0.02) (Table 4). Additionally, among the 54 cases in the fentanyl group, nine cases that had already developed IVH grade 3–4 prior to fentanyl administration were excluded to appropriately evaluate the effect of fentanyl use. Consequently, 45 cases were included in the final analysis. In this analysis, 31.1% (14/45 cases) in the fentanyl group developed IVH grade 3–4, compared to 23.8% (20/84 cases) in the non-fentanyl group. However, the difference between the two groups was not statistically significant (*p* = 0.34). These findings suggest that fentanyl use may not directly impact the incidence of IVH grade 3–4. Further studies with larger sample sizes and multivariate analyses are warranted to explore potential confounding factors and refine the interpretation of these results.

**Table 4:** Effect of fentanyl on the risk of tension pneumothorax after excluding specific factors.

Analysis Group	Adjusted odds ratio (95% CI)	*p*-value
Excluding PPHN cases	0.07 (0.01–0.67)	0.02
Excluding CAM stage 2–3 cases	0.04 (0.002–0.71)	0.03
Excluding non-tertiary hospital birth	0.11 (0.01–0.99)	0.05
Excluding oligohydramnios cases	0.07 (0.008–0.67)	0.02

CAM, chorioamnionitis; CI, confidence interval; PPHN, persistent pulmonary hypertension of the newborn.

Sensitivity analyses using different caliper widths for propensity score matching (0.2 SD and 0.3 SD) yielded consistent results. At both caliper widths, fentanyl use remained significantly associated with a reduced risk of tension pneumothorax (OR: 0.10, 95% CI: 0.01–0.75, *p* = 0.03; OR: 0.10, 95% CI: 0.01–0.61, *p* = 0.02).

### Secondary analyses and covariates

Further secondary analyses explored the effects of fentanyl infusion rate and duration on tension pneumothorax risk. The median fentanyl infusion rate was 1.5 μg/kg/h (range: 0.5–3.0), and the median duration was 72 h (range: 6–120). Exploratory analyses revealed a trend toward a protective effect with fentanyl infusion rates, although this association did not reach statistical significance (Coefficient = −1.60, *p* = 0.25). Similarly, fentanyl infusion duration showed no meaningful association with the outcome (Coefficient = −0.008, *p* = 0.95). For secondary variables, 16 cases (29.6%) required ventilator mode changes from SIMV to HFOV. However, estimation of the association between ventilator mode changes and tension pneumothorax risk failed due to complete separation resulting from low event counts, preventing valid statistical modeling. Additionally, nine cases (16.7%) required surfactant re-administration, but the analysis was not feasible due to sparse data, which limited estimation and interpretation. Covariate analysis indicated a non-significant trend toward reduced risk with SIMV (Coefficient = −5.01, *p* = 0.16). However, antenatal steroid administration (Coefficient = 3.13, *p* = 0.43) and CAM stage 2–3 (Coefficient = −2.84, *p* = 0.45) were not significantly associated with tension pneumothorax risk.

In the cohort-style sensitivity analysis, balance improved after IPTW across all prespecified covariates, with SMDs reduced to below 0.1 (Supplementary Table S2, Figure S1). Across different estimation methods, fentanyl exposure was consistently associated with a reduced risk of TP within 72 h (Crude OR: 0.10, 95% CI: 0.01–0.75; Firth OR: 0.12, 95% CI: 0.01–0.54; IPTW OR: 0.05, 95% CI: 0.003–0.76; AIPW OR: 0.06, 95% CI: 0.009–0.37), whereas the overlap weighting model showed a similar trend with wide confidence intervals (OR: 0.04, 95% CI: 0.001–2.37). In contrast, no significant associations were observed between fentanyl exposure and mortality before discharge (ORs ranging: 1.14–1.32 across methods) or severe IVH (ORs ranging: 1.49–1.79), with confidence intervals consistently spanning unity (Supplementary Table S3).

## Discussion

This study demonstrated a significant association between fentanyl use and a lower incidence of tension pneumothorax in extremely preterm neonates, even after adjustment using propensity score matching. Subgroup analyses further supported the robustness of these findings, as fentanyl maintained a protective effect in groups excluding specific high-risk conditions such as PPHN, CAM, and oligohydramnios. Sensitivity analyses with varying caliper widths also confirmed the consistency of these results. An air leak in the lungs is associated with the rupture of an overdistended alveolus that may develop due to generalized air trapping or an uneven gas distribution (21). Fentanyl, a potent synthetic *mu*-opioid receptor agonist, is commonly used for sedation and analgesia in infants (22, 23). The administration of fentanyl infusion in preterm infants ventilated for hyaline membrane disease significantly reduced behavioral sedation scores, O_2_ desaturation, and the neuroendocrine stress response (18). It is plausible that sedation might alleviate distress by reducing the amount of time that the baby is awake and by reducing struggles related to awareness of a noxious environment. In addition, the latter effect may reduce adverse respiratory and other outcomes by reducing undesirable interactions between the infant and ventilator breathing (24). Previous reports have identified potential risk factors for tension pneumothorax, including oligohydramnios, CAM stage 2–3, out-of-hospital birth, and PPHN. Furthermore, it has been hypothesized that the use of antenatal steroids mitigates this risk (25–27). A multivariate analysis was conducted incorporating respiratory settings such as VG mode, SIMV, and HFOV; however, no significant differences were observed in these parameters (28, 29). In the present multivariate analysis, we revealed that the use of fentanyl may reduce the risk of tension pneumothorax within 72 h after birth. Fentanyl controls respiration appropriately and promotes the synchronization of ventilation; therefore, it may have prevented tension pneumothorax in our study. Randomized controlled trials (RCTs) have been conducted to examine the incidence of air leak with sedative use: Quinn's study compared the incidence between morphine and pancuronium usage groups in infants born at 24–34 weeks of gestation (30), Wood and colleagues compared the incidence of morphine and diamorphine use in infants born at 26–30 weeks of gestation (31), and Lago and colleagues compared the incidence between the fentanyl use and non-use infants born at 29–33 weeks of gestation (18). None of these studies found a significant difference in the air leak incidence; however, they did not include infants born at 22–23 weeks of gestation and were not RCTs specifically comparing fentanyl use and non-use. This retrospective case-control study suggests that the use of fentanyl may reduce the incidence of tension pneumothorax within 72 h of birth in extremely preterm infants born at 22–23 weeks of gestation. However, given the retrospective design and the absence of randomization or blinding, the potential for information bias and recall bias cannot be entirely excluded.

Nevertheless, all infants born at 22–23 weeks of gestation in Kagoshima Prefecture were admitted to a tertiary-level NICU, minimizing the risk of selection bias. To further address potential confounding factors, propensity score matching was employed to achieve a balance between the fentanyl-exposed and non-exposed groups. Additionally, multivariate analyses were performed to enhance the robustness and reliability of the results.

Subgroup analyses demonstrated that the protective effect of fentanyl remained consistent even after excluding high-risk groups, such as those with PPHN, CAM stage 2–3, and oligohydramnios. Furthermore, sensitivity analyses using different caliper widths for propensity score matching (0.2 SD and 0.3 SD) confirmed the consistency and reproducibility of the findings, reinforcing the association between fentanyl use and a lower incidence of tension pneumothorax. In addition, a cohort-style sensitivity analysis using IPTW and Firth logistic regression achieved good covariate balance and showed results in the same protective direction for fentanyl, although the confidence intervals remained wide due to the rarity of events. These findings suggest that the protective association is robust but should be interpreted with caution. In a study by Lago and colleagues comparing the use of fentanyl (0.5–2 µg/kg/h, with an average infusion duration of 72 h) vs. non-use, involving infants born at 29–33 weeks of gestation, it was reported that behavioral and neuroendocrine stress responses were reduced; however, no effect was observed on the duration of mechanical ventilation or tolerance to enteral feeding (18). After birth, extremely preterm infants are exposed to painful and stressful situations, such as endotracheal intubation, oral gastric tube insertion, venous and arterial catheter insertion, management of ventilation, and repeated heel lances. Based on the limited data available, the capacity for the conscious perception of pain can arise only after the thalamocortical pathways begin to function, which may occur in the third trimester at around 29–30 weeks of gestation. Small-scale histological studies on human fetuses have shown that thalamocortical fibers begin to form at 23–30 weeks of gestation (32). Additional analyses in this study suggested a non-significant trend toward a protective effect related to fentanyl infusion rate and duration. However, analyses of ventilator mode changes and surfactant re-administration were limited by sparse data and complete separation, preventing valid estimation. These findings highlight the need for larger studies to validate the observed protective effects of fentanyl use against tension pneumothorax in extremely preterm neonates.

Neurophysiological afferent pain pathways reach the cortex between weeks 20 and 26. Although thalamocortical fibers are necessary for pain perception, this pathway must also be functional. Although changes in pain behavior can be observed with gestational age, even very premature neonates show behavioral, physiological, and hormonal stress responses to painful stimuli (33). Opioids for analgesia and sedation, such as morphine and fentanyl, are started as continuous infusions to provide comfort while on mechanical ventilation, reduce pain and stress, and potentially reduce neurological injury in infants born at 22–23 weeks. A Cochrane systematic review has shown that opioids have little or no effect on neurodevelopmental outcomes at 18–24 months; similarly, they do not affect the duration of mechanical ventilation, rate of neonatal mortality, or incidence of intraventricular hemorrhage (either any grade or grade 3/4) (34). However, the authors did not include studies focusing on infants born at 22–23 weeks and those with tension pneumothorax. It is desirable to conduct an RCT focusing on infants born at 22–23 weeks of gestation stratified into groups based on opioid exposure and non-exposure, which could be used to compare the effects of opioid usage and non-usage on mortality, morbidity (tension pneumothorax, IVH, necrotizing enterocolitis, and hypotension), duration of mechanical ventilation, and neurodevelopment.

## Conclusion

This study has shown that using fentanyl may reduce the risk of tension pneumothorax within 72 h of birth in infants born at 22–23 weeks of gestation. However, its retrospective design and potential biases limit the generalizability of these findings. Future research should involve RCTs and longitudinal studies to explore mortality rates and neurodevelopmental outcomes, helping inform clinical practice for managing preterm infants.

## Data Availability

The original contributions presented in the study are included in the article/Supplementary Material, further inquiries can be directed to the corresponding author.
