# Supplementary figures and images for: Association between fentanyl use and reduced risk of tension pneumothorax in extremely preterm infants born at 22–23 weeks' gestation: a retrospective case–control study
Source: Front Pediatr. 2025 Oct 3;13:1643333. doi: 10.3389/fped.2025.1643333 (PMC12531072; doi:10.3389/fped.2025.1643333)

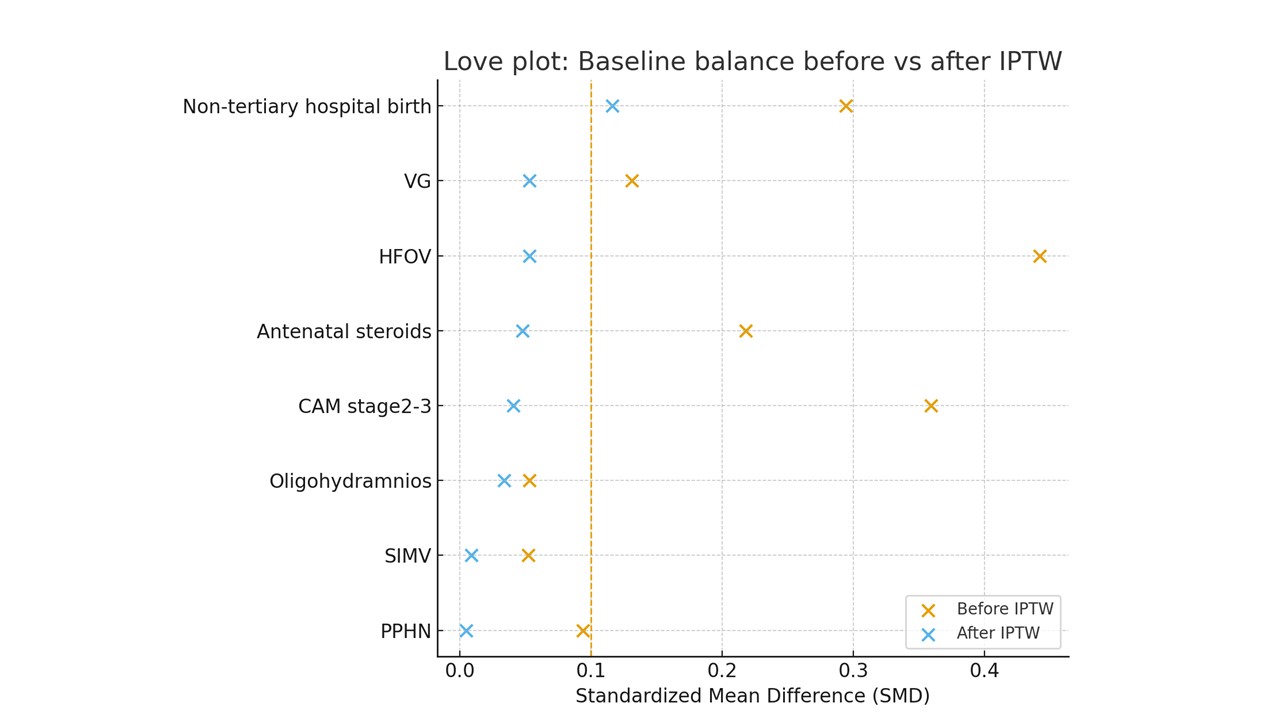

Supplement: Supplementary file 1 [file Image1.jpeg]
