# Supplementary material for: Association between fentanyl use and reduced risk of tension pneumothorax in extremely preterm infants born at 22–23 weeks' gestation: a retrospective case–control study
Source: Front Pediatr. 2025 Oct 3;13:1643333. doi: 10.3389/fped.2025.1643333 (PMC12531072; doi:10.3389/fped.2025.1643333)
Supplement: Supplementary file 2 [file Datasheet1.pdf]

Figure legend

Figure S1 Covariate balance (Love plot) comparing fentanyl and non-fentanyl groups before and after inverse probability of treatment weighting (IPTW)

Table

Supplementary Table 1. Comparison of baseline characteristics between fentanyl and non-fentanyl groups

	**Fentanyl use (n=54) % or IQR**	**Non-fentanyl use (n=84) % or IQR**	**p value**
**22w**	14 (25.9)	28 (33.3)	0.45
**Sex (male)**	27 (50)	43 (51.2)	1
**Birth weight (median)**	561 (470-611)	532 (475-592)	0.82
**FGR (10%tile)**	9 (16.7)	9 (10.7)	0.31
**Survival discharge**	35 (64.8)	57 (67.9)	0.72
**Death (less than 1 week)**	6 (11.1)	7 (8.3)	0.77
**Non-tertiary hospital birth**	8 (14.8)	5 (6)	0.13
**Maternal age**	32 (28-34)	30 (27-35)	0.16
**C/S**	47 (87)	59 (70.2)	0.02
**Antenatal steroids**	29 (53.7)	36 (42.9)	0.23
**Tocolysis**	39 (72.2)	52 (61.9)	0.27
**MgSO4**	29 (53.7)	39 (46.4)	0.49
**Antibiotics**	15 (27.8)	23 (27.4)	1
**PROM**	17 (31.5)	30 (35.7)	0.71
**CAM stage2-3**	17 (31.5)	41 (48.8)	0.052
**Funisitis stage2-3**	12 (22.2)	18 (21.4)	1

**HDP**	4 (7.4)	0	0.02
**GDM**	1 (1.9)	4 (4.8)	0.65
**HELLP**	3 (5.6)	0	0.06
**Oligohydramnios**	2 (3.7)	4 (4.8)	1
**Fertility treatment**	7 (13)	7 (8.3)	0.4
**Abruption**	8 (14.8)	5 (6)	0.13
**Placenta previa**	0	1 (1.2)	1
**MD twin**	2 (3.7)	8 (9.5)	0.32
**APS1min**	2 (1-3)	2 (1-3)	0.34
**APS5min**	6 (4-7)	6 (4-7)	0.46
**UApH**	7.33 (7.24-7.36)	7.32 (7.25-7.38)	0.51
**RDS**	54 (100)	84 (100)	1
**TP**	1 (1.9)	15 (17.9)	0.005
**IVH Grade3-4**	23 (42.6)	20 (23.8)	0.02
**Cystic PVL**	4 (7.4)	5 (6)	0.74
**NEC**	6 (11.1)	5 (6)	0.34
**FIP**	10 (18.5)	8 (9.5)	0.19
**MRI**	3 (5.6)	2 (2.4)	0.38
**PDA surgery**	2 (3.7)	8 (9.5)	0.32
**EOS**	0	3 (3.6)	0.55
**PPHN**	6 (11.1)	7 (8.3)	0.77
**SIMV**	48 (88.9)	76 (90.5)	0.78
**HFOV**	18 (33.3)	46 (54.8)	0.02
**VG**	26 (48.2)	35 (41.7)	0.49
**PB**	18 (33.3)	20 (23.8)	0.25

**DEX**	7 (13)	4 (4.8)	0.11
**MDZ**	6 (11.1)	3 (3.6)	0.15
**Re-intubation**	1 (1.9)	4 (4.8)	0.65
**Cardiac massage**	2 (3.7)	0	0.15
**Retreatment of surfactant**	9 (16.7)	12 (14.3)	0.81
**Fetal bradycardia**	4 (7.4)	5 (6)	0.74

Abbreviations: APS, Apgar score; CAM, chorioamnionitis; C/S, cesarean section; CI, confidence interval; DEX, dexmedetomidine; EOS, early-onset sepsis; Fertility treatment, including AIH (artificial insemination with husband), ICSI (intracytoplasmic sperm injection), and IVF-ET (in vitro fertilization and embryo transfer); FGR, fetal growth restriction; FIP, focal intestinal perforation; GA, gestational age; GDM, gestational diabetes mellitus; HDP, hypertensive disorders; of pregnancy; HFOV, high-frequency oscillatory ventilation; IQR, interquartile range; IVH, intraventricular hemorrhage; MD twin, monochorionic diamniotic twin; MDZ, midazolam; MRI, meconium related ileus; $MgSO_4$, magnesium sulfate; NEC, necrotizing enterocolitis; PB, phenobarbital; PDA, patent ductus arteriosus; PPHN, persistent pulmonary hypertension of the newborn; PROM, premature rupture of membranes; PVL, periventricular leukomalacia; RDS, respiratory distress syndrome; SIMV, synchronized intermittent mandatory ventilation; TP, tension pneumothorax; UApH, umbilical artery pH; VG, volume guarantee ventilation.

Supplementary Table 2. Covariate balance between fentanyl-exposed and non-exposed groups before and after IPTW

Variable	Exposed% (unweighted)	Unexposed% (unweighted)	SMD (unweighted)	Exposed % (IPTW)	Unexposed% (IPTW)	SMD (IPTW)
**PPHN**	11.1	8.3	0.094	9.6	9.4	0.005
**SIMV**	88.9	90.5	0.052	91.4	91.1	0.009
**HFOV**	33.3	54.8	0.442	44.7	47.3	0.053
**VG mode**	48.1	41.7	0.131	46.9	44.3	0.053
**Non-tertiary hospital birth**	14.8	6	0.294	11.8	8.3	0.116
**Oligohydramnios**	3.7	4.8	0.053	5.5	4.8	0.034
**Antenatal steroids**	53.7	42.9	0.218	49.1	46.8	0.048
**CAM stage2-3**	31.5	48.8	0.359	40.7	42.7	0.041

Abbreviations: CAM, chorioamnionitis; CI, confidence interval; HFOV, high-frequency oscillatory ventilation; IPTW, inverse probability of treatment weighting; PPHN, persistent pulmonary hypertension of the newborn; SIMV, synchronized intermittent mandatory ventilation; SMD, standardized mean difference; VG, volume guarantee.

Supplementary Table 3. Adjusted and comparative effect estimates for the association between fentanyl use and clinical outcomes

Table 3a. Adjusted odds ratios from Firth logistic regression for tension pneumothorax within 72 h after birth

Variable	Odds ratio（95%CI）	p-value
**Fentanyl use**	0.12 (0.013-0.54)	0.003
**PPHN**	0.72 (0.06-4.35)	0.74
**SIMV**	0.44 (0.06-2.84)	0.36
**HFOV**	0.49 (0.13-1.68)	0.26
**VG mode**	1.21 (0.31-4.75)	0.78
**Non-tertiary hospital birth**	3.24 (0.57-18.9)	0.18
**Oligohydramnios**	4.71 (0.73-26.8)	0.097
**Antenatal steroids**	0.41 (0.10-1.46)	0.17
**CAM stage2-3**	1.83 (0.59-5.90)	0.29

Abbreviations: CAM, chorioamnionitis; CI, confidence interval; HFOV, high-frequency oscillatory ventilation; PPHN, persistent pulmonary hypertension of the newborn; SIMV, synchronized intermittent mandatory ventilation; VG, volume guarantee.

Table 3b. Adjusted odds ratios from Firth logistic regression for in-hospital mortality

**Variable**	**Odds ratio（95%CI）**	**p-value**
**Fentanyl use**	1.14 (0.53-2.46)	0.73
**PPHN**	2.69 (0.83-9.01)	0.098
**SIMV**	1.06 (0.34-3.70)	0.92
**HFOV**	1.43 (0.66-3.15)	0.37
**VG mode**	1.12 (0.49-2.57)	0.79
**Non-tertiary hospital birth**	2.02 (0.57-7.13)	0.27
**Oligohydramnios**	3.89 (0.83-22.9)	0.084
**Antenatal steroids**	0.72 (0.32-1.59)	0.42
**CAM stage2-3**	0.84 (0.39-1.80)	0.66

Abbreviations: CAM, chorioamnionitis; CI, confidence interval; HFOV, high-frequency oscillatory ventilation; PPHN, persistent pulmonary hypertension of the newborn; SIMV, synchronized intermittent mandatory ventilation; VG, volume guarantee.

Table 3c. Adjusted odds ratios from Firth logistic regression for severe intraventricular hemorrhage (grade 3–4)

Variable	Odds ratio（95%CI）	p-value
**Fentanyl use**	1.79 (0.77-4.24)	0.18
**PPHN**	3.11 (0.85-11.9)	0.087
**SIMV**	3.85 (0.88-25.7)	0.075
**HFOV**	1.19 (0.50-2.87)	0.69
**VG mode**	2.37 (0.95-6.21)	0.063
**Non-tertiary hospital birth**	0.24 (0.023-1.21)	0.087
**Oligohydramnios**	0.62 (0.06-3.62)	0.61
**Antenatal steroids**	0.43 (0.17-1.02)	0.055
**CAM stage2-3**	0.54 (0.22-1.24)	0.15

Abbreviations: CAM, chorioamnionitis; CI, confidence interval; HFOV, high-frequency oscillatory ventilation; PPHN, persistent pulmonary hypertension of the newborn; SIMV, synchronized intermittent mandatory ventilation; VG, volume guarantee.

Table 3d. Summary of crude and adjusted effect estimates (Crude, Firth, IPTW, Overlap weighting, AIPW) for primary and secondary outcomes

Variable	Crude OR	Firth OR	Overlap OR	IPTW OR	AIPW OR
**TP**	0.10(0.01-0.75)	0.12(0.01-0.54)	0.04(0.001-2.37)	0.047(0.003-0.76)	0.06(0.009-0.37)
**Mortality**	1.32(0.63-2.78)	1.14(0.53-2.46)	1.30(0.43-3.94)	1.19(0.57-2.50)	1.21(0.56-2.61)
**IVH Grade 3-4**	1.49(0.68-3.26)	1.79(0.77-4.24)	1.54(0.48-4.97)	1.69(0.79-3.64)	1.69(0.74-3.85)

Abbreviations: CI, confidence interval; AIPW, augmented inverse probability weighting; IPTW, inverse probability of treatment weighting; IVH, intraventricular hemorrhage; OR, odds ratio; SE, standard error; TP, tension pneumothorax within 72 hours after birth.

Note: Values in parentheses indicate 95% CI.
